# Impact of Personalized Avatars and Motion Synchrony on Embodiment and Users’ Subjective Experience: Empirical Study

**DOI:** 10.2196/40119

**Published:** 2022-11-08

**Authors:** Myeongul Jung, Sangyong Sim, Jejoong Kim, Kwanguk Kim

**Affiliations:** 1 Department of Computer Science Hanyang University Seoul Republic of Korea; 2 Department of Psychology Duksung Woman’s University Seoul Republic of Korea

**Keywords:** embodiment, virtual reality, virtual avatar, personalization, personalized, body motion, presence, simulator sickness, simulator, simulation, avatar, motion, body ownership, self location, agency, experience, virtual world, immersive

## Abstract

**Background:**

Embodiment through a virtual avatar is a key element for people to feel that they are in the virtual world.

**Objective:**

This study aimed to elucidate the interaction between 2 methods of eliciting embodiment through a virtual avatar: motion synchronization and appearance similarity between a human and avatar, to understand embodiment (agency, body ownership, and self-location) and subjective experience (presence, simulator sickness, and emotion) in virtual reality.

**Methods:**

Using a full-body motion capture system, 24 participants experienced their virtual avatars with a 3D-scanned face and size-matched body from a first-person perspective. This study used a 2 (motion; sync and async) × 2 (appearance; personalized and generic) within-subject design.

**Results:**

The results indicated that agency and body ownership increased when motion and appearance were matched, whereas self-location, presence, and emotion were affected by motion only. Interestingly, if the avatar’s appearance was similar to the participants (personalized avatar), they formed an agency toward the avatar’s motion that was not performed by themselves.

**Conclusions:**

Our findings would be applicable in the field of behavioral therapy, rehabilitation, and entertainment applications, by eliciting higher agency with a personalized avatar.

## Introduction

Recent developments in technology have made humans citizens of the virtual world (widely known as the “metaverse”). People use virtual reality (VR) devices including head-mounted displays (HMDs) to wander around the digital world. Additionally, people use an entity such as a virtual avatar in VR to overcome limitations that their physical body cannot achieve. However, it is difficult for people to feel that they are the owner of this newly created body. This feeling of owning the body is called embodiment [1]. Embodiment consists of 3 components: body ownership (feeling that the body undergoing a certain experience is subordinate to oneself [2]), agency (feeling that “I” am the cause for body motion [2]), and self-location (“My view is located at the place where it should be” [1]).

Previous studies have found that it is possible to form a sense of embodiment toward external objects such as a rubber hand [3], virtual arm [4], and virtual body [5]. To induce this illusory feeling of embodiment, several studies [1,5-8] have used a motion-capture device for measuring a body movement and an HMD for observing a virtual avatar’s actions with first-person perspective to synchronize a human activity with the movement of the virtual avatar. These studies showed that motion synchrony increased the degree of agency and found that people felt an ownership toward the virtual body used in the experiments. In addition, increased agency could make people feel a greater presence with less simulator sickness in VR [7]. Various studies have used the motion synchronization method to induce size perception [6] and emotion [5], as well as reduce racial bias [9].

Other studies have developed a method of enhancing embodiment by increasing appearance similarity between a real body and virtual body [10-12]. For instance, Kim et al [11] demonstrated a body size–matching method using 4 measurements of height, shoulder width, belly width, and hip width. Likewise, Gorisse et al [10] attempted to increase appearance similarity by attaching a 3D-scanned head to a virtual body, and furthermore, Waltemate et al [12] presented a “personalized avatar” by attaching a 3D-scanned head to a participant-sized body. These studies found that the more an avatar’s body resembled a human’s, the more people felt ownership of the virtual avatar’s body [10-12]. This increment in ownership resulted in higher presence [11,12] and decreased simulator sickness [11], which could increase people’s virtual experience. In line with this, Jung et al [8] showed that it is possible to measure one’s body-related perception by using a virtual body resembling the individual’s physical characteristics.

Although embodiment can be enhanced with motion synchrony (factor 1) and appearance similarity (factor 2), there has been little research to discover the interaction effect between these 2 factors. Kim et al [1] demonstrated that the 2 factors affected body ownership independently using a dot avatar and body size–matched avatar. Moreover, they found that the factors affecting the subcomponents of embodiment could boost each other, which can be beneficial to future applications of VR. As we can create more personalized avatars using technologies such as 3D scanning [12-14], it is worth investigating whether the boosting effect between the 2 factors would be maintained with more individualized avatars.

This study aimed to detect the effects of 2 factors (motion synchrony and appearance similarity) on embodiment and their secondary effect on virtual experiences. Our first hypothesis is that motion synchronization would increase the feeling of agency and body ownership toward the virtual body. The second hypothesis is that the appearance similarity would increase body ownership toward the virtual body. We also speculate that there are potential interactions between the 2 factors. Third, in the higher embodiment condition, the participants would have a more positive subjective experience toward VR comprising presence, simulator sickness, and emotion.

## Methods

### Participants

In all, 24 participants (age: mean 23.29, SD 1.97 years; female: n=12, 50%) were recruited for the study. A power analysis using PASS power analysis and sample size software (version 2019; NCSS) was run to determine sample size. All participants provided written informed consent and were compensated with US $40 for their cooperation.

### Ethics Approval

All experimental protocols were approved by the Hanyang University Institutional Review Board (HYUIRB-202107-014-1). Photographed individuals in the figures provided written informed consent to publish the case details.

### Hardware Setup

To generate synchrony between the participant and avatar, a motion-capture system (Motive; version 2.0.2; Natural Point) and 18 Flex 13 cameras (Natural Point) were used. Participants wore a full-body motion-capture suit with 37 reflective markers. To generate a 3D-scanned face, images of the participant’s face were captured using a mobile device (iPhone SE2; Apple Inc). With the captured images, the participant’s 3D face model was generated through the Metashape (Agisoft LLC) and Blender (Blender Institute) software. The virtual environment of the experiment was implemented using Unity software (version 2018.3.0f2; Unity Technologies). The software was run on a desktop PC (Windows 10 OS; Microsoft) with Intel Core i7-6700 (Intel) CPU, 16GB RAM (Samsung), and NVIDIA GeForce GTX 3070 (Nvidia) GPU.

### Virtual Environment

**Figure 1:**
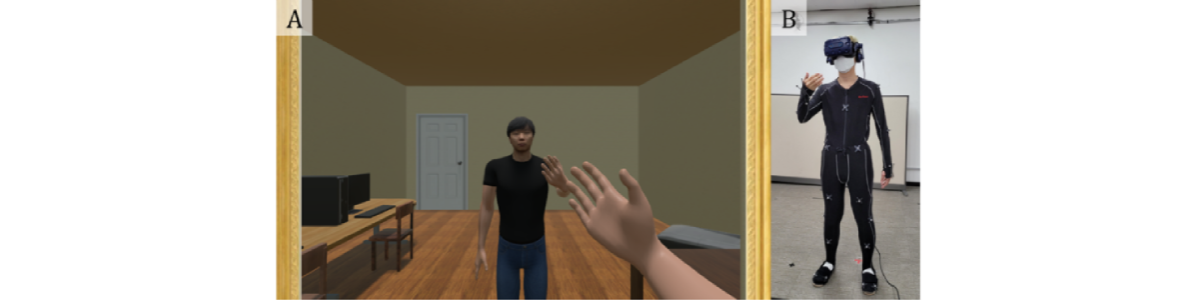
Virtual environment (A) and experimental settings (B) of this study. (A) illustrates the virtual environment from the participant’s perspective. A virtual mirror was set in front of the participant’s location. (B) illustrates the experimental setting of each participant, where each participant wears a motion-capture suit with 37 reflective markers attached and a head-mounted display for observing their virtual avatar’s movement in first-person perspective.

Participants wore a motion-capture suit and HMD (HTC Vive Pro eye; HTC) to experience the virtual environment. The virtual environment was a small room, about 4 m (width) × 4 m (length) × 2.5 m (height), and a virtual mirror was set in front of the participant’s location. A participant could observe the virtual avatar’s body either directly through first-person perspective or by looking in the mirror. The virtual environment and experimental settings are presented in Figure 1.

### Procedures

#### Avatar Creation Phase

**Figure 2:**
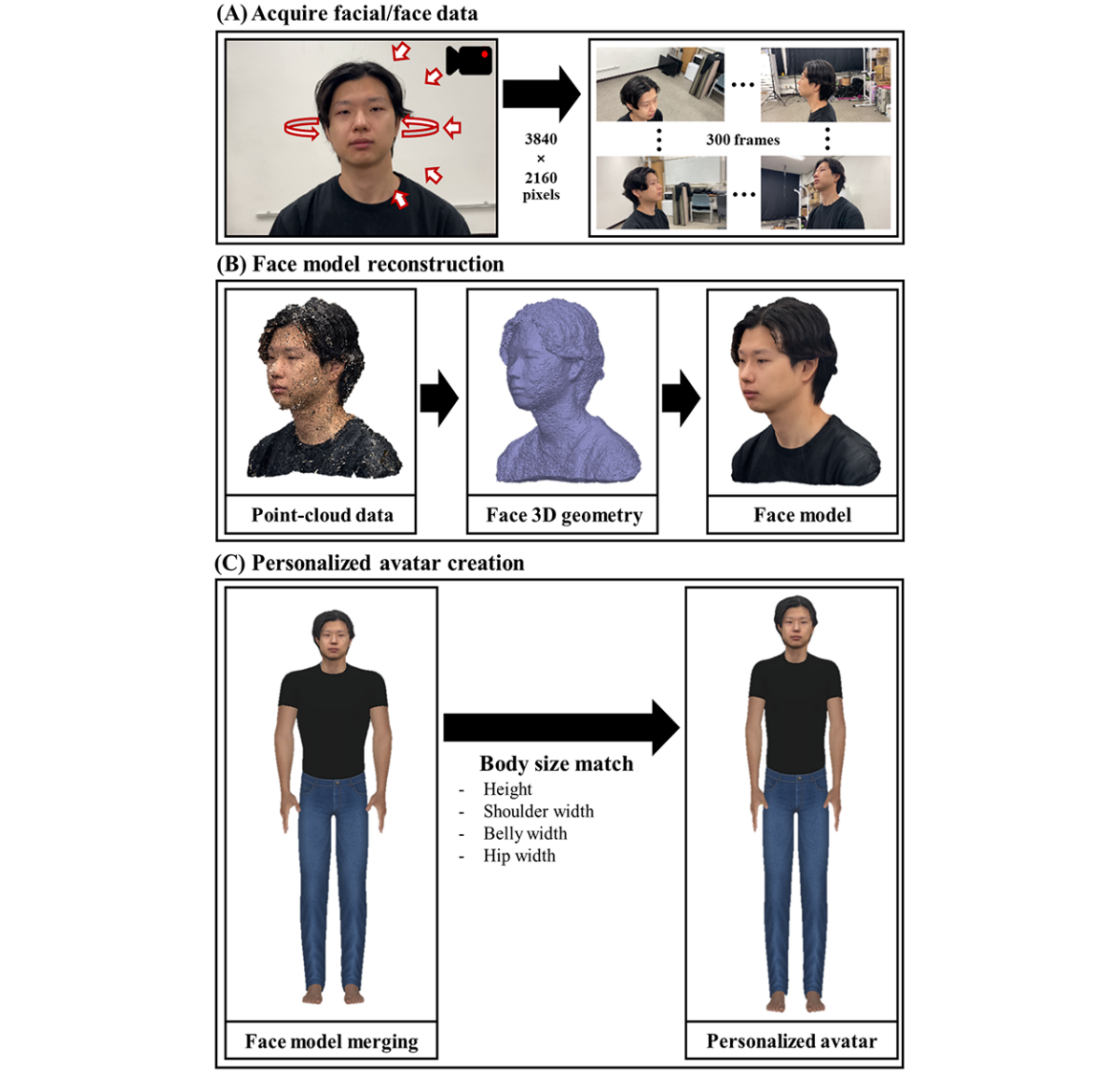
Procedure for avatar creation. (A) 300 facial image data of diverse angles in 4K resolution are acquired through a mobile device. (B) Point-cloud data are extracted from the images and processed into a face model by the Metashape program. (C) Each participant’s face model is merged with an avatar body, and the avatar’s body size is adjusted to that of the participant.

In this phase, the personalized avatar was created through the 3D scanning of participants’ faces. Before the scanning, each participant completed the questionnaires for demographic information. After completing the questionnaires, the experimenter measured the participant’s height, shoulder width, belly width, and hip width. They then underwent face scanning, where the experimenter recorded the participant’s face while rotating around them in diverse angles for approximately 1 minute. A total of 300 frame images (3840 × 2160 pixels of resolution) of the participant’s face in different angles were selected. Subsequently, images were processed by the Metashape program to build 3D point-cloud data. The point-cloud data were then simplified into 3D geometry and texture of each participant’s face, and then their 3D face model was merged with an existing virtual avatar body. Finally, the participants’ body sizes were applied to a virtual avatar’s body to produce a personalized avatar. Figure 2 shows the process of the avatar creation.

#### Experimental Phase

**Figure 3:**
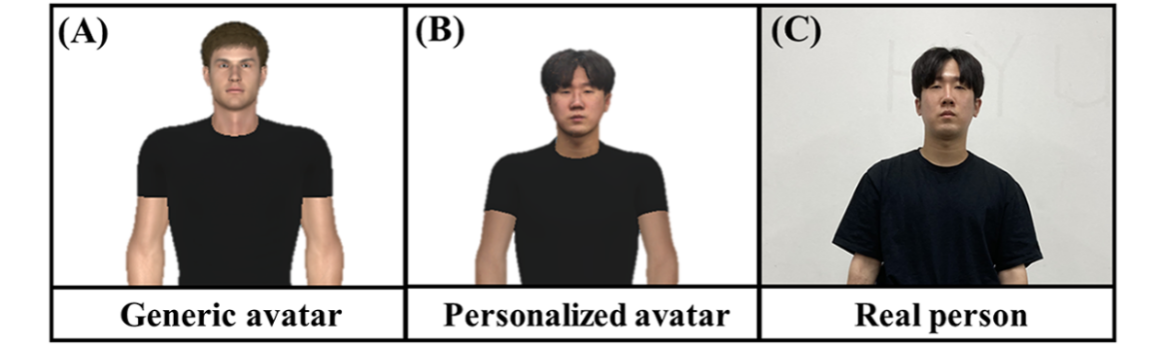
Example of a (A) generic avatar and (B) personalized avatar for the (C) real person.

This experiment had a 2 (motion; sync vs async) × 2 (appearance; personalized vs generic) within-subject design to examine the main effects of motion synchrony and appearance similarity and the interaction effect between them. In the motion “sync” condition, the virtual avatar’s body moved according to the participant’s movement, whereas in the motion “async” condition, the virtual avatar moved according to prerecorded movements, regardless of the participant’s movement. For appearance similarity (or personalized appearance), a virtual avatar consisting of a participant’s 3D-scanned head with a size- and gender-matched body was used. In the generic appearance condition, only a gender-matched avatar was used. Figure 3 shows examples of personalized and generic avatars used for a male participant.

During the experimental phase, participants underwent 4 blocks of VR experiences (ie, sync-personalized, sync-generic, async-personalized, and async-generic); the order of the blocks was counterbalanced using the Latin-square method. Participants were asked to move freely and observed a virtual avatar for 5 minutes during each block. After each block, the participants completed the similarity, embodiment, and virtual experience questionnaires listed below. Between blocks, participants rested until they felt ready to start the next block. The entire procedure lasted approximately 90 minutes.

### Dependent Measures

#### Similarity Questionnaire

The Similarity Questionnaire (SQ) from a recent study [13] was applied in this study. The SQ consists of four 7-point Likert scale questions assessing participants’ feeling on how similar the body parts of the virtual avatar are akin to theirs. The scale ranges from –3 (fully disagree) to +3 (fully agree); Table 1 shows the 4 questions in detail. The average score of the questions was used as the similarity score.

**Table 1:** List of items used in the Similarity Questionnaire^a^.

Question	Statement
Face	I felt the face of virtual body was similar to mine.
Torso	I felt the torso of virtual body was similar to mine.
Arms	I felt the arm of virtual body was similar to mine.
Legs	I felt the leg of virtual body was similar to mine

^a^Scale ranges from –3 (fully disagree) to +3 (fully agree).

#### Embodiment Questionnaire

The Embodiment Questionnaire (EQ) from recent studies [1,5,6,8,11] was modified and used in this study. The EQ consists of three 7-point Likert scale questions on agency, body ownership, and self-location. The EQ scale ranges from –3 (fully disagree) to +3 (fully agree). Each question is detailed in Table 2.

**Table 2:** List of items used in the Embodiment Questionnaire^a^.

Question	Statement
Agency	I felt that the movement of the virtual body was caused by my own movements.
Body ownership	I felt that the virtual body I saw when looking at myself in the mirror was my own body.
Self-location	I felt that I was inside the virtual body.

^a^Scale ranges from –3 (fully disagree) to +3 (fully agree).

#### Virtual Experience Questionnaire

The Virtual Experience Questionnaire consisted of 3 subjective virtual experiences: presence, simulator sickness, and emotion. Participants’ presence was assessed with the Presence Questionnaire [15], which consists of 21 questions using a 7-point Likert scale ranging from 1 (not at all) to 7 (completely). Participants’ simulator sickness was assessed with the Simulator Sickness Questionnaire [16], which has 16 questions for checking symptoms. Each question was scored using a 4-point Likert scale ranging from 0 (not at all) to 3 (severe). Participants’ emotions were assessed with the self-assessment manikin [17]. It consists of 2 subscales of arousal and valence. A visual representation of a manikin with varying levels of emotional expression was used to rate participants’ emotional arousal, from 1 (extremely calm) to 9 (extremely excited), and valence, from 1 (extremely negative) to 9 (extremely positive).

### Data Analysis

All data were analyzed using SPSS statistical software (version 27.0; IBM Corp). The evaluations of normality were performed using the skewness, kurtosis, and Kolmogorov-Smirnov tests. A 2 (sync vs async) × 2 (personalized vs generic) repeated measures ANOVA was conducted on all dependent measures to examine the effects of motion synchrony, appearance similarity, and interaction between the 2 variables. Post hoc analyses then followed. The level of statistically significant *P* value was set to .05.

## Results

### Similarity

The average similarity score from the SQ did not show a significant main effect for motion (*F*_1,23_=1.448; *P*=.24; sync mean .14; async mean –.01), whereas a significant main effect was demonstrated for appearance (*F*_1,23_=32.920; *P*<.001; η^2^=.589; personalized mean .92; generic mean –.79). The interaction effect between the 2 factors was not significant (*F*_1,23_=1.178; *P*=.29).

### Embodiment

The result of embodiment is illustrated in Table 3.

**Table 3:** Embodiment scores of agency, body ownership, and self-location.

Embodiment, appearance		Motion		*P* value
		Sync, mean (SE)	Async, mean (SE)	
**Agency**				
	Personalized	2.38 (.13)	–.79 (.42)	<.001
	Generic	2.25 (.22)	–1.75 (.36)	<.001
	Sync (personalized vs generic)	N/A^a^	N/A	.59
	Async (personalized vs generic)	N/A	N/A	.02
**Body ownership**				
	Personalized	1.54 (.20)	.00 (.41)	<.001
	Generic	.96 (.33)	–1.08 (.42)	.002
	Sync (personalized vs generic)	N/A	N/A	.08
	Async (personalized vs generic)	N/A	N/A	.01
**Self-location**				
	Personalized	1.58 (.26)	–.29 (.42)	<.001
	Generic	1.25 (.34)	–.29 (.42)	<.001
	Sync (personalized vs generic)	N/A	N/A	.22
	Async (personalized vs generic)	N/A	N/A	>.99

^a^N/A: not applicable.

#### Agency

The agency score displayed a significant main effect for motion (*F*_1,23_=91.653; *P*<.001; η^2^=.799) and appearance (*F*_1,23_=4.442; *P*=.046; η^2^=.162). A significant interaction effect was present between the 2 factors (*F*_1,23_=5.867; *P*=.02; η^2^=.203). Post hoc analysis revealed that the participants reported higher agency in the sync condition than the async condition, regardless of appearance (personalized-sync vs personalized-async: *P*<.001; generic-sync generic-async: *P*<.001). Although the virtual avatar moved regardless of a participant’s body motion in the async condition, participants felt higher agency toward the personalized avatar’s movement (*P*=.02).

#### Body Ownership

The body ownership score exhibited a significant main effect for motion (*F*_1,23_=22.876; *P*<.001; η^2^=.499) and appearance (*F*_1,23_=10.047; *P*=.004; η^2^=.312). The interaction effect between the 2 factors was not significant (*F*_1,23_=1.078; *P*=.31).

#### Self-location

The self-location score showed a significant main effect for motion (*F*_1,23_=31.306; *P*<.001; η^2^=.576); however, no main effect for appearance was found (*F*_1,23_=.416; *P*=.53). There was no significant interaction effect between the 2 factors (*F*_1,23_=.358; *P*=.56).

### Virtual Experience

The result of virtual experience from the Virtual Experience Questionnaire is illustrated in Table 4.

**Table 4:** Virtual experience scores of presence, simulator sickness, and emotion (arousal and valence).

Virtual experience, appearance		Motion		*P* value
		Sync, mean (SE)	Async, mean (SE)	
**Presence**				
	Personalized	74.21 (2.42)	55.71 (3.68)	<.001
	Generic	74.71 (3.01)	54.92 (4.00)	<.001
	Sync (personalized vs generic)	N/A^a^	N/A	.77
	Async (personalized vs generic)	N/A	N/A	.71
**Simulator sickness**				
	Personalized	23.53 (5.62)	24.00 (5.46)	.86
	Generic	25.09 (6.18)	27.12 (6.29)	.52
	Sync (personalized vs generic)	N/A	N/A	.58
	Async (personalized vs generic)	N/A	N/A	.43
**Arousal**				
	Personalized	4.13 (.39)	4.08 (.40)	.86
	Generic	3.96 (.41)	3.96 (.33)	>.99
	Sync (personalized vs generic)	N/A	N/A	.57
	Async (personalized vs generic)	N/A	N/A	.61
**Valance**				
	Personalized	6.38 (.27)	5.63 (.37)	.004
	Generic	5.75 (.31)	5.54 (.29)	.41
	Sync (personalized vs generic)	N/A	N/A	.07
	Async (personalized vs generic)	N/A	N/A	.81

^a^N/A: not applicable.

#### Presence

The presence score showed a significant main effect for motion (*F*_1,23_=40.126; *P*<.001; η^2^=.636) but not for appearance (*F*_1,23_=.009; *P*=.93). Furthermore, the interaction effect between the 2 factors was not significant (*F*_1,23_=.333; *P*=.57).

#### Simulator Sickness

The main effects of motion (*F*_1,23_=.247; *P*=.62) and appearance were not significant (*F*_1,23_=.608; *P*=.44). Additionally, the interaction effect between the 2 factors was not significant (*F*_1,23_=.269; *P*=.61).

#### Emotion

Arousal did not indicate a significant main effect for motion (*F*_1,23_=.023; *P*=.88) or appearance (*F*_1,23_=.495; *P*=.49). There was no significant interaction effect between the 2 factors (*F*_1,23_=.016; *P*=.90).

Valence showed a significant main effect for motion (*F*_1,23_=6.111; *P*=.02; η^2^=.210) and no main effect for appearance (*F*_1,23_=1.325; *P*=.26). There was no significant interaction effect between the 2 factors (*F*_1,23_=3.524; *P*=.07).

## Discussion

### Principal Findings

This study investigated the effects of motion synchronization and appearance similarity on participants’ embodiment, perceived similarity, and subjective experience in VR. The results showed that participants experienced a higher level of agency and body ownership when body motion was synchronized and appearance was similar to theirs. Surprisingly, under the motion and appearance synchronizations, participants reported that the body motion of the virtual avatar was driven by them even when the virtual avatar moved independently, suggesting that the personalized appearance of the virtual avatar’s body can create an illusory agency toward the avatar’s movement that was not performed by the participant. Furthermore, our results indicated that the synchronization of motion could contribute to higher presence and induce more positive emotion.

Two novel findings on embodiment can be listed from the results of this study. First, there was a statistically significant interaction between motion and appearance on agency (significant difference between async-personalized and async-generic conditions in agency). Prior studies have reported that synchronizing motion [1,5-7] could contribute to form a higher level of agency. Results from this study support such previous findings, proving that the integration of visual-motion synchronization, which fulfills proprioception [18], forms a feeling that “I am the only cause of the avatar’s body motion” [1]. In addition to this finding, this study demonstrated that agency was associated with body motion that was not caused by the participant; therefore, the body motion could be felt as the participant’s movement when the virtual avatar resembled the participant’s appearance. This kind of attribution of behavior toward oneself has been shown in several previous studies. For example, Aymerich-Franch et al [19] reported that participants felt shame and guilt for the misbehavior of a humanoid robot, which they did not perform. Likewise, Jun et al [5] reported that participants’ emotions changed to happy, neutral, and sad according to a virtual avatar’s emotional status. Furthermore, this study extends previous findings that body movement can be attributed to oneself with a personalized avatar. In the motion sync condition, however, the agency difference was not significant between the personalized and generic avatars. We speculate that the reason behind this finding is a higher agency score (average score of 2.31 out 3), which might have caused a ceiling effect. The current result can be used in the field of cognitive behavioral therapy, such as behavior modeling, which involves observing others to learn desirable behaviors. Using a personalized avatar, which facilitates more agency toward the avatar’s action, can be more effective compared to using general avatars in behavior modeling.

Second, it was evident that the 2 factors (ie, motion and appearance) in this study affected body ownership independently. Prior studies have found that synchronizing motion [11] and personalized avatars [1,10-12] could contribute to form a higher level of body ownership. The current findings support previous studies by showing that the integration of motion synchronization [1] and visual aspects with higher fidelity [10] form a feeling that the body is one’s own. Prior studies have revealed that the level of visual fidelity increased in the order of point-light avatar [1], robot avatar [10], human avatar [5], size matched avatar [11], and 3D-scanned avatar [12]. This study extends these previous findings by revealing that body ownership can be further increased with a avatar that has a 3D-scanned face and size-matched body.

In addition, it was evident that the factors of subjective virtual experience (ie, presence, simulator sickness, and emotion) were affected by motion synchrony. This result supports previous studies that confirmed higher presence [1,11] and positive emotion [1,5] by motion synchronization. Furthermore, a moderate interaction between appearance similarity (personalized vs generic) and motion synchrony (sync vs async) in emotional valence (significant difference between sync and async in personalized avatar, whereas no difference in generic avatar condition in valence; Table 4) showed that motion synchronization with a personalized avatar could induce more positive emotions than a generic avatar. On the contrary, the effect on arousal and simulator sickness was not significant. Low arousal scores across the conditions suggest that the participants were in a calm state during the experimental tasks. We speculate that low arousal might have affected simulator sickness, resulting in a floor effect across conditions.

In addition to the main findings addressed above, it is worth noting the methodology we used for generating personalized avatars in this study. In fact, many prior studies have attempted to create personalized avatars by 3D scanning a participant’s face and body [2,10,12,14]. However, due to complex settings such as red-green-blue-depth camera [14] or multiple time-synchronized red-green-blue cameras [12,13], it is challenging to use this methodology to construct a personalized avatar; thus, it is not appropriate for general use. Inspired by Gorisse et al [10] and Jung et al [8], our study proposed a more convenient method for creating a personalized avatar using a single smartphone and body-size measuring. The result from the SQ showed that our method could enhance a feeling of appearance similarity between the person and avatar. Thus, in future metaverse applications, each user can easily generate their personalized avatar using their smartphone to dive into VR.

There are a few limitations in this study. First, the participants were restricted to healthy young adults. Future studies should consider recruiting participants across diverse age groups and health statuses. Second, as the prerecorded animation of the async condition consisted of simple body movements that can be performed easily by the participants, it would be stimulating to use a body motion that is “impossible” to perform to generalize the effect of appearance similarity on agency in the follow-up studies. Third, this study asked participants to move freely in the main task and measured arousal and valence as emotional variables. Although appearance similarity did not affect participants’ emotions, future studies should consider participants who have negative feelings toward their body, such as individuals with eating disorders [20]. An attitude toward one’s body should be considered for future medical applications.

### Conclusion

This study proposed a method for creating personalized avatars using a single smartphone camera and an avatar’s body size manipulation. Furthermore, this study’s result indicated that participants perceived that the virtual avatar’s appearance was similar to them. Furthermore, participants established a higher sense of embodiment toward the virtual avatar’s body that was similar to theirs in body motion and appearance. Moreover, we discovered that the synchronization of appearance could result in a sense of agency toward an avatar’s movement. We hope that the findings in the current study can contribute to the fields of physical activity promotion [21], social cognition training [22], and pain intervention [23], suggesting that matching body motion and appearance can enhance the VR experience.

## Abbreviations

EQ: Embodiment Questionnaire
HMD: head-mounted display
SQ: Similarity Questionnaire
VR: virtual reality
